# Dimethyl Sulfoxide Damages Mitochondrial Integrity and Membrane Potential in Cultured Astrocytes

**DOI:** 10.1371/journal.pone.0107447

**Published:** 2014-09-19

**Authors:** Chan Yuan, Junying Gao, Jichao Guo, Lei Bai, Charles Marshall, Zhiyou Cai, Linmei Wang, Ming Xiao

**Affiliations:** 1 Jiangsu Province Key Laboratory of Neurodegeneration, Department of Anatomy, Nanjing Medical University, Nanjing, Jiangsu, China; 2 Department of Rehabilitation Sciences, University of Kentucky Center For Excellence in Rural Health, Hazard, Kentucky, United States of America; 3 Department of Neurology, the Affiliated Hospital of Anhui Medical University, Lu'an People's Hospital, Lu'an, Anhui, China; University of Nebraska Medical Center, United States of America

## Abstract

Dimethyl sulfoxide (DMSO) is a polar organic solvent that is used to dissolve neuroprotective or neurotoxic agents in neuroscience research. However, DMSO itself also has pharmacological and pathological effects on the nervous system. Astrocytes play a central role in maintaining brain homeostasis, but the effect and mechanism of DMSO on astrocytes has not been studied. The present study showed that exposure of astrocyte cultures to 1% DMSO for 24 h did not significantly affect cell survival, but decreased cell viability and glial glutamate transporter expression, and caused mitochondrial swelling, membrane potential impairment and reactive oxygen species production, and subsequent cytochrome c release and caspase-3 activation. DMSO at concentrations of 5% significantly inhibited cell variability and promoted apoptosis of astrocytes, accompanied with more severe mitochondrial damage. These results suggest that mitochondrial impairment is a primary event in DMSO-induced astrocyte toxicity. The potential cytotoxic effects on astrocytes need to be carefully considered during investigating neuroprotective or neurotoxic effects of hydrophobic agents dissolved by DMSO.

## Introduction

Dimethyl sulfoxide (DMSO) is a polar organic solvent with various biological functions that is used extensively in both biological and medical research [1], [2]. In the neuroscience field, DMSO is commonly used to dissolve hydrophobic neuroprotective or neurotoxic agents [3]. Studies have also investigated the potential effects of DMSO itself on the nervous system [4]–[8]. DMSO treatment has been shown to reduce infarction volume and neuronal loss in rodent cerebral ischemia models [9]–[11]. Nevertheless, it has been demonstrated that DMSO produces widespread neuronal apoptosis in the developing mouse brain and neuronal loss in rat hippocampal culture [12]. DMSO also can induce tau hyperphosphorylation, a hallmark of Alzheimer's disease, in the adult mouse brain [13]. DMSO produces a dose-dependent reduction in nerve conduction velocity with myelin disruption of the rat sciatic nerve [4]. These discrepant data suggest that DMSO plays protective or injurious roles in the brain, which may be due to differences in the model used as well as the concentration of DMSO administered.

Furthermore, there are several case reports that reveal severe neurotoxicity [14]–[18], encephalopathy [19]–[21] and transient global amnesia [22] associated with the infusion of DMSO-cryopreserved hematopoietic stem/progenitor cells. These results highly suggest that DMSO has neurological side effects and toxicity. Therefore, it is necessary to further explore the neuropharmacological and neurotoxicological effects of DMSO.

Astrocytes, the most numerous glial cells in the mammalian brain, are responsible for maintaining brain homeostasis [23]. For example, astrocytes regulate glutamatergic neurotransmission and prevent glutamate excitotoxicity by removing excess glutamate from the extracellular synaptic space through glutamate transporters [24]. Astrocyte dysfunction has been implicated in a variety of neurological diseases [25]. Based on this, it is necessary to investigate effects of DMSO on astrocytes. However, to our knowledge, this issue has not been explored.

In the present study we provided evidence that DMSO has cytotoxic effects on astrocytes via disruption of mitochondrial integrity and membrane potential, resulting in oxidative stress, apoptosis and down-regulation of glutamate transporters. This finding expands our understanding of the neurotoxic mechanisms of DMSO and provides an experimental basis to select a safe dose of DMSO in neuroscience research.

## Results

### DMSO decreased survival and viability of cultured astrocytes

**Figure 1:**
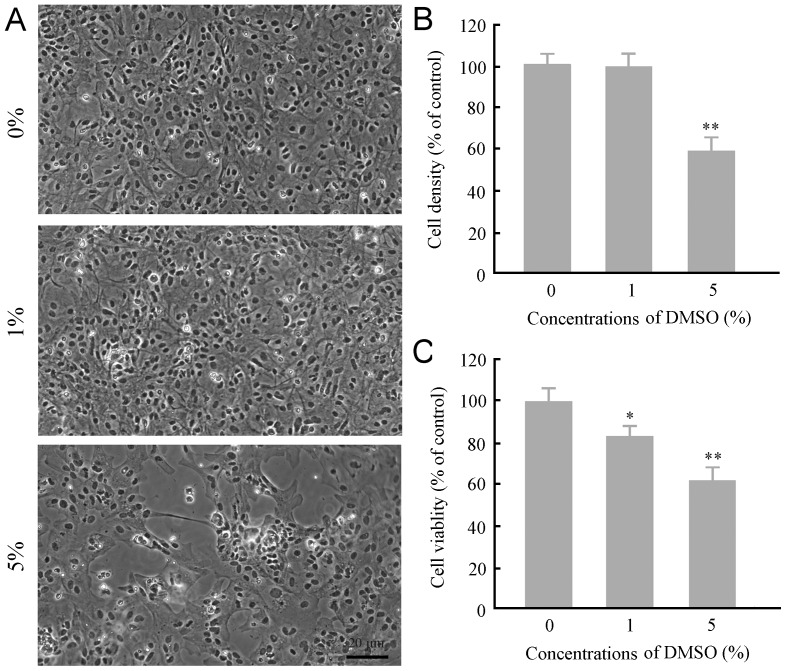
Effects of DMSO on survival and viability of mouse cortical astrocytes in culture. (**A**) General growth profile of incubated with various concentrations of DMSO for 24 h. Astrocytes were grown well and closely adhered to each other on the surface of coverslips under normal circumstances and exposed to 1% DMSO. Many astrocytes were detached from the coverslips in 5% DMSO culture medium. (**B–C**) Quantitative analysis of the cell density (B) and cell viability (C) of astrocytes incubated with different concentrations of DMSO for 24 h. Data are shown as a mean ± SEM of five independent experiments performed in triplicate. *P<0.05 and **P <0.01 versus control group.

DMSO at concentrations of 0.5–1.5% is widely used as a solvent for various pharmacological agents in neuroscience research [3]. Thus, we selected DMSO at concentrations of 1% and 5% to observe and compare their neurotoxic profile in cultured astrocytes during a 24-h exposure period. Exposure of cultures to 1% DMSO had no significant effect on the growth and survival of astrocytes, but the cell viability was decreased by 16%, as revealed by 3-(4,5-Dimethylthiazol-2-yl)-2,5-diphenyltetrazolium bromide (MTT) assay. DMSO at concentrations of 5% caused a 40% decrease in the cell density and a 32% decrease in the cell viability (Figure 1A–C).

### DMSO impaired mitochondrial integrity and membrane potential of cultured astrocytes

**Figure 2:**
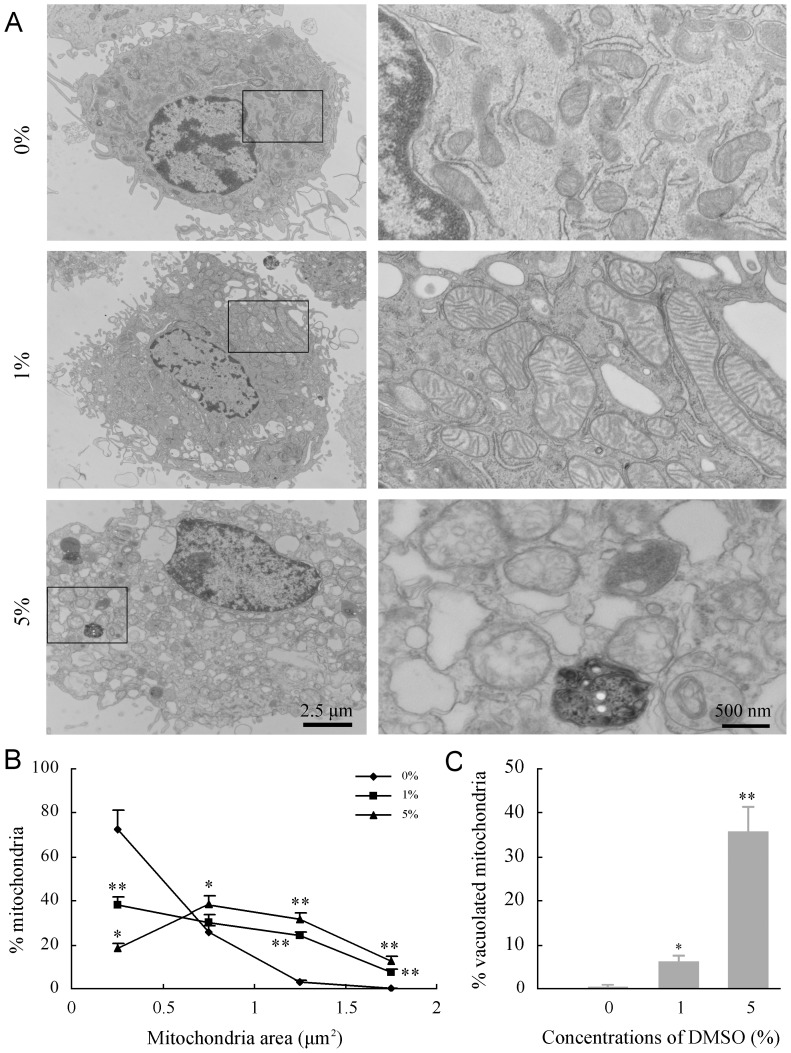
Effects of DMSO on substructures of cultured mouse cortical astrocytes. (**A**) Representative transmission electron micrographs showing substructural morphology of astrocytes after treatment with different concentrations of DMSO for 24 h. The disruption of mitochondria integrity becomes more severe with increased DMSO concentrations. Fragmentation of the nucleus, with condensation and margination of nuclear chromatin, was frequently observed in astrocytes exposed to 5% DMSO. (**B**) Quantitation of mitochondrial cross-sectional area. The results confirmed DMSO-induced mitochondrial swelling, with a significant rightward shift in the mitochondrial area cumulative frequency curve, relative to untreated control. (**C**) The quantitative analysis showed increases in the percentage of mitochondrial vacuolization in astrocytes treated with DMSO in a dose-dependent manner. Data are shown as a mean ± SEM of five independent experiments. *P<0.05 and **P<0.01 versus control group.

DMSO has been shown to concentration-dependently modulate the structure and properties of cell membranes [26], [27]. The mitochondrial membrane has a typical lipid bilayer structure and is vulnerable to various injury factors [28]. It is also known that mitochondrial damage is a major cause of decreased cell viability [29]. Thus, the impairment of DMSO on astrocyte mitochondria was investigated by using the transmission electron microscopy. Astrocytes treated with 1% DMSO for 24 h showed swelling of mitochondria (Figure 2A), as revealed by quantitation of mitochondrial cross-sectional area (Figure 2B). The swelling and disruption of mitochondria were more evident after exposure to 5% DMSO, and about 35% of mitochondria exhibited loss of cristae or formed monolayer vacuoles (Figure 2C).

**Figure 3:**
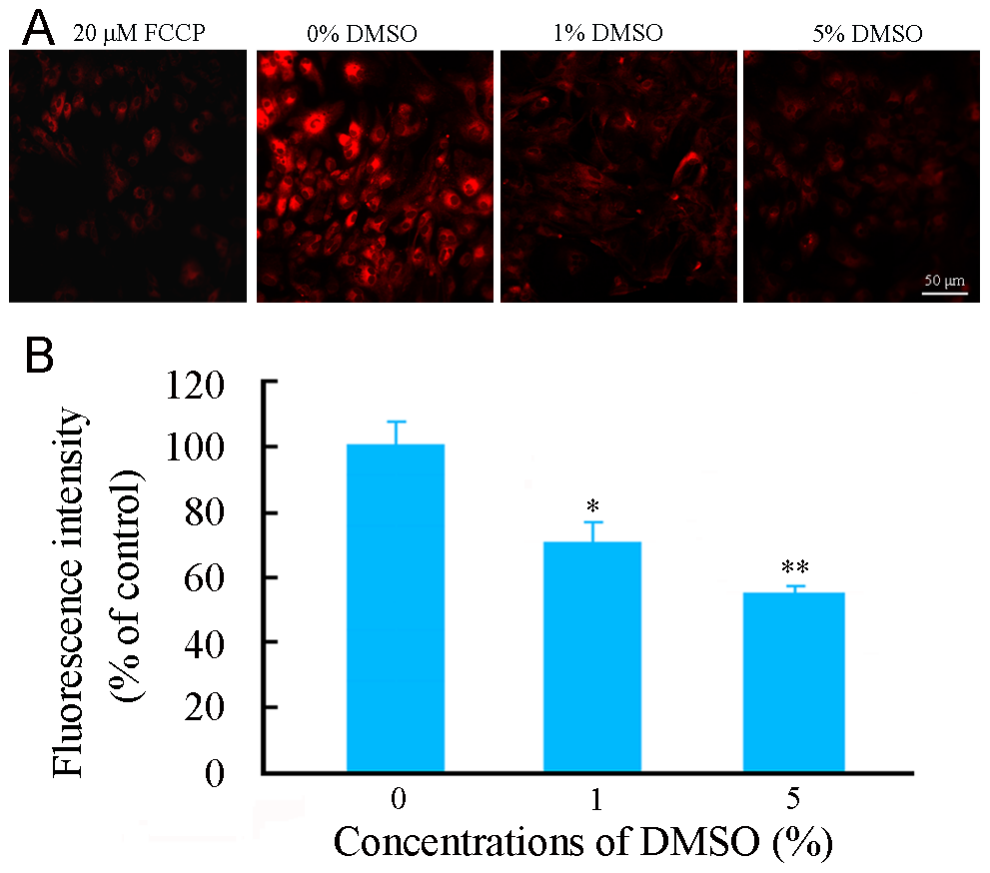
Effects of DMSO on ΔΨm of cultured mouse cortical astrocytes. (**A**) Representative micrographs showing ΔΨm, revealed by TMRE fluorescent staining, in astrocytes treated with various concentrations of DMSO for 24 h. The left first micrograph is a positive control for depolarized mitochondria by incubated with 20 µM FCCP, an uncoupler of electron transport and oxidative phosphorylation, for 10 minutes prior to staining with TMRE. (**B**) The quantitative analysis revealed that TMRE fluorescence intensity was decreased in astrocytes treated with DMSO in a dose-dependent manner. Data are shown as a mean ± SEM of five independent experiments performed in triplicate. *P<0.05 and **P<0.01 versus control group.

The deleterious effect of DMSO on mitochondrial function of astrocytes was also determined by measurement of mitochondrial membrane potential (ΔΨm) using fluroescence microscopy of tetramethylrhodamine methyl (TMRE). DMSO at concentrations in 1% and 5% caused significant decreases in ΔΨm in cultured astrocytes, as shown in Figure 3A and B.

### DMSO caused mitochondrial cytochrome c (Cyt c) release in cultured astrocytes

**Figure 4:**
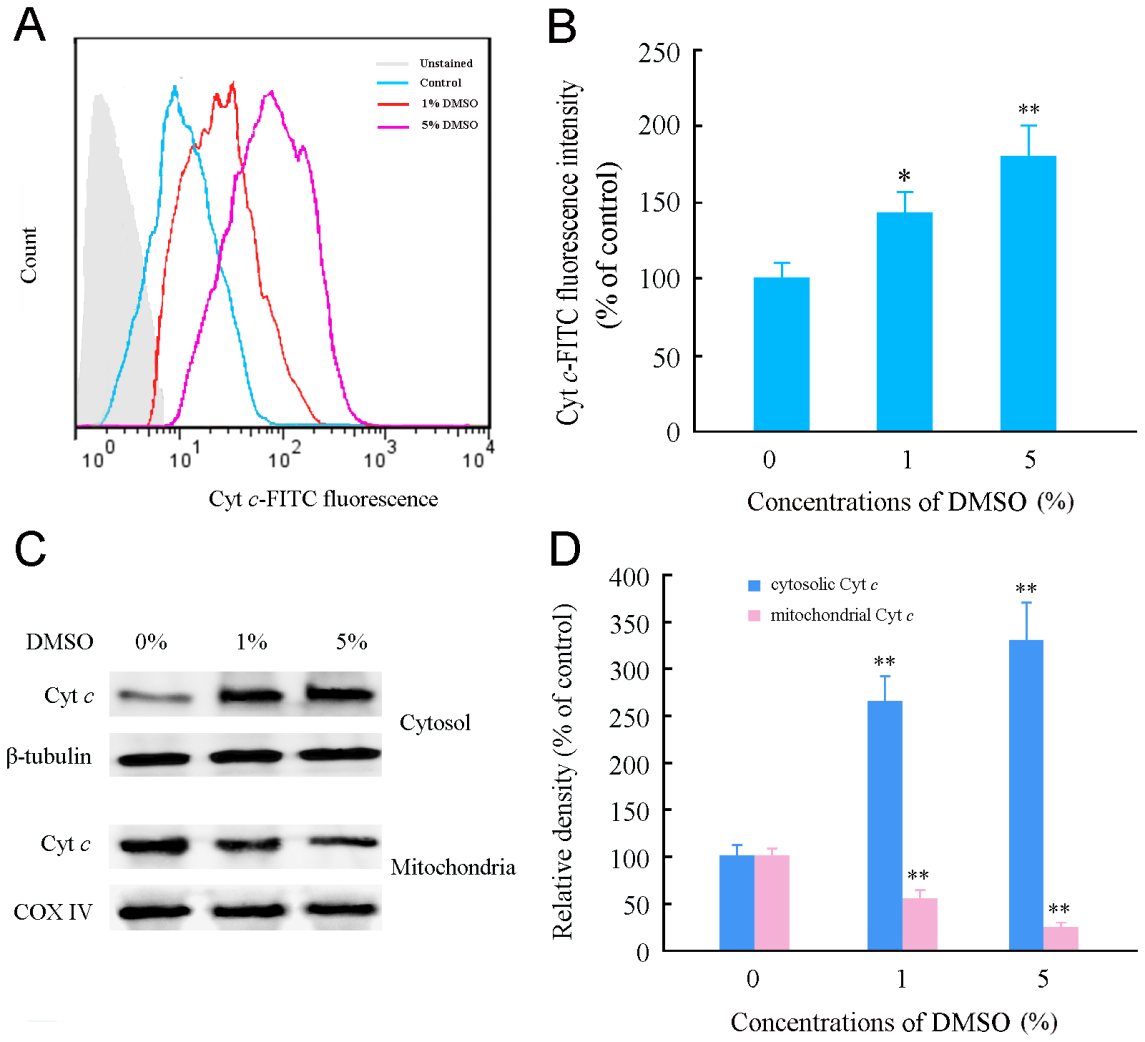
Effects of DMSO on and the release of mitochondrial Cyt c and intracellular ROS generation in cultured mouse cortical astrocytes. (A) Representative flow cytometry data showing mitochondrial Cyt *c*-FITC fluorescence within the cytoplasm of astrocytes treated with various concentrations of DMSO for 24 h. (B) Quantitative analysis of Cyt *c*-FITC fluorescence intensity. (C) Representative western blot bands showing expression levels of Cyt *c* in cytosolic and mitochondrial fractions of astrocytes treated with various concentrations of DMSO for 24 h. β-tubulin and COX IV, which were exclusively expressed within the cytosol and mitochondria, respectively, were used as loading controls. (D) The quantitative analysis of the relative optical density of cytosol and mitochondrial Cyt *c* showing that DMSO caused translocation of Cyt *c* from the mitochondria into the cytoplasm of astrocytes. Data are shown as a mean ± SEM of five (for flow cytometry) or four (for Western blot) independent experiments performed in triplicate. *P<0.05 and **P<0.01 versus control group.

It is also known that disruption of mitochondrial integrity results in a release of Cyt c from the mitochondrial intermembrane space into the cytosol [30]. Therefore, we investigated the effects of DMSO on mitochondrial Cyt c release in cultured astrocytes. The flow cytometry results showed that the concentration of Cyt c in the astrocyte cytoplasm increased after treatment with 1% and 5% DMSO for 24 h (Figure 4A–B). The Western blotting further confirmed that DMSO induced Cyt c release from the mitochondria. DMSO-treated astrocytes had low levels of Cyt c in the mitochondrial fraction and high levels of Cyt c in the cytosolic fraction, compared with intact controls (Figure 4C–D).

### DMSO increased reactive oxygen species (ROS) generation in cultured astrocytes

**Figure 5:**
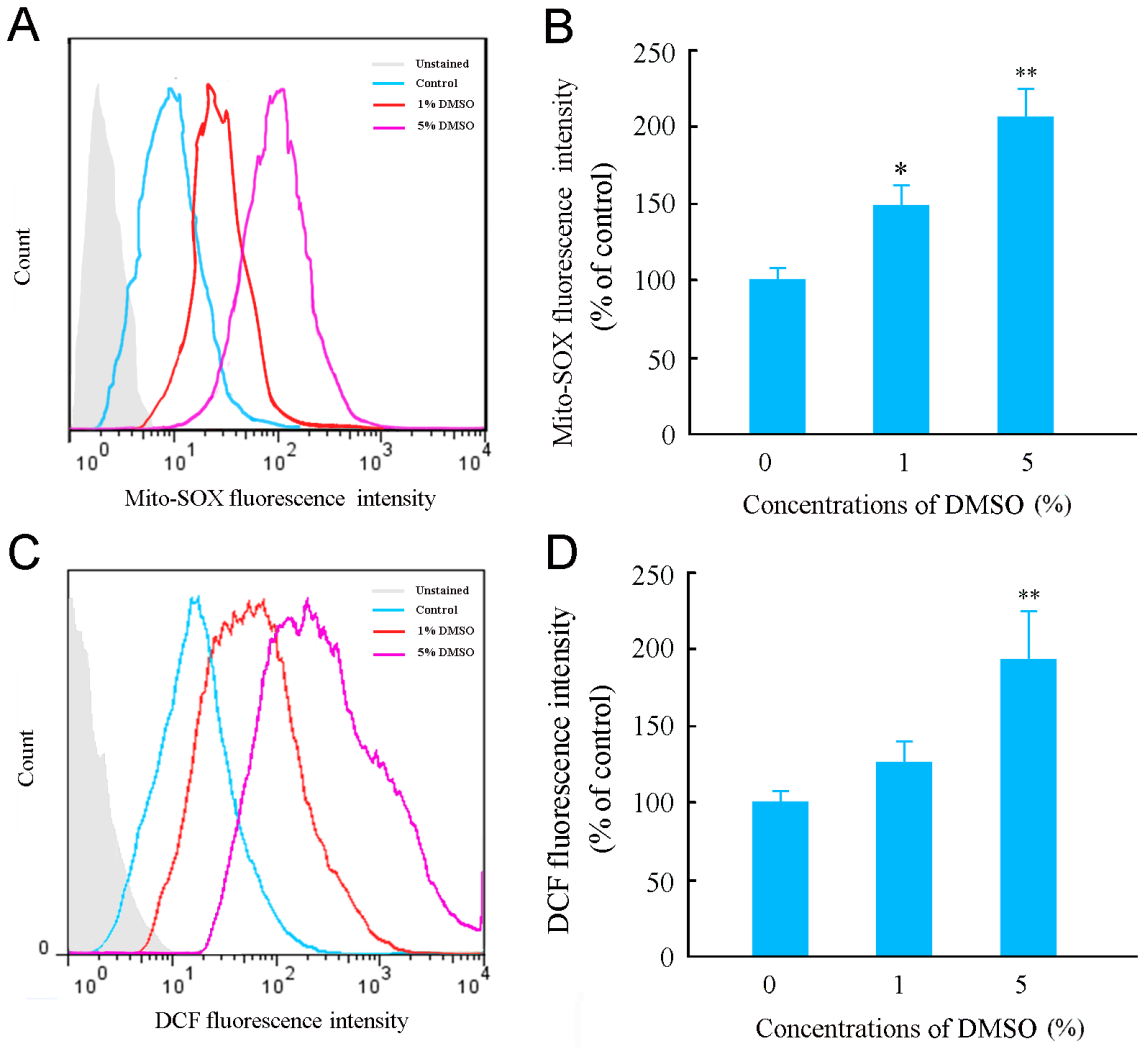
Effects of DMSO on mitochondrial and intracellular ROS generation in cultured mouse cortical astrocytes. (**A**) Representative flow cytometry data showing Mito-SOX fluorescence, a highly selective indicator of superoxide in live cell mitochondria, in astrocytes treated with various concentrations of DMSO for 24 h. (**B**) Quantitative analysis revealed that the Mito-SOX fluorescence intensity increased in astrocytes treated with DMSO with a dose-dependent manner. (**C**) Representative flow cytometry data showing DCF fluorescence in astrocytes treated with various concentrations of DMSO for 24 h. (**D**) The quantitative analysis showed that ROS levels were increased in astrocytes treated with DMSO at 5% concentration but not at 1%, by detecting the fluorescence intensity of DCF. (**E**) Data are shown as a mean ± SEM of five independent experiments performed in triplicate. *P<0.05, **P<0.01 versus control group. #*P*<0.05, ## *P*<0.01 versus 5% DMSO treated group.

It has been demonstrated that decreased ΔΨm reduces aerobic metabolism and increases ROS generation [31], [32]. Thus, the generation of mitochondrial ROS in DMSO-treated astrocytes was assessed by detection of MitoSOX-Red, a highly selective detector of superoxide in live cell mitochondria, using the flow cytometry. The intensity of Mito-SOX fluorescence was increased in a concentration dependent manner (Figure 5A–B). DMSO-increased intracellular ROS levels were also observed using an oxidant-sensitive probe DCFH-DA and the flow cytometry (Figure 5C–D).

### DMSO induced apoptosis of cultured astrocytes

**Figure 6:**
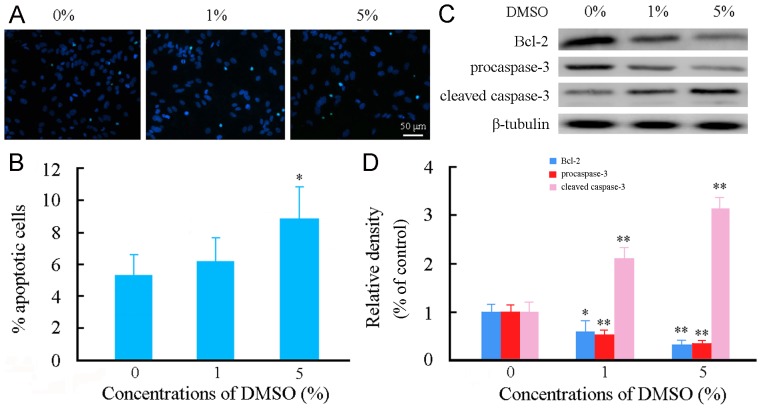
Effects of DMSO on apoptosis of cultured mouse cortical astrocytes. Astrocytes were incubated with various concentrations of DMSO for 24 h. (A) Representative micrographs showing TUNEL positive apoptotic astrocytes (cyan-blue). Cell nuclei counterstained with Hoechst 33342 (blue). (B) Quantitative analysis of astrocyte apoptosis. (C) Representative western blot bands showing expression levels of procaspase-3, cleaved caspase-3 and Bcl-2 in astrocytes. (D) The quantitative analysis showed that Bcl-2 and procaspase-3 expression levels were decreased, but cleaved caspase-3 expression level was increased in astrocytes treated with 1% or 5% DMSO. Data are shown as a mean ± SEM of five (for TUNEL) or four (for Western blot) independent experiments performed in triplicate. *P<0.05 and **P<0.01 versus control group.

There is evidence that Cyt *c* release and ROS generation caused by mitochondria damage and/or dysfunction play a central role in initiating apoptosis [33]–[36]. Thus, we examined the effects of DMSO on astrocyte apoptosis. The terminal deoxynucleotidyl transferase dUTP nick end labeling (TUNEL) and quantitative analysis revealed that the percentage of apoptotic astrocytes was not significantly different between the baseline condition and 1% DMSO, but increased in 5% DMSO (Figure 6A–B). Astrocytes exposed to 1% or 5% DMSO for 24 h showed decreases in anti-apoptotic protein Bcl-2 expression and procaspse-3 expression, while increases in cleaved caspase-3 expression, as revealed by the Western blot analysis (Figure 6C–D).

### DMSO decreased glutamate transporter 1 (GLT-1) and glutamate-aspartate transporter (GLAST) expression in cultured astrocytes

**Figure 7:**
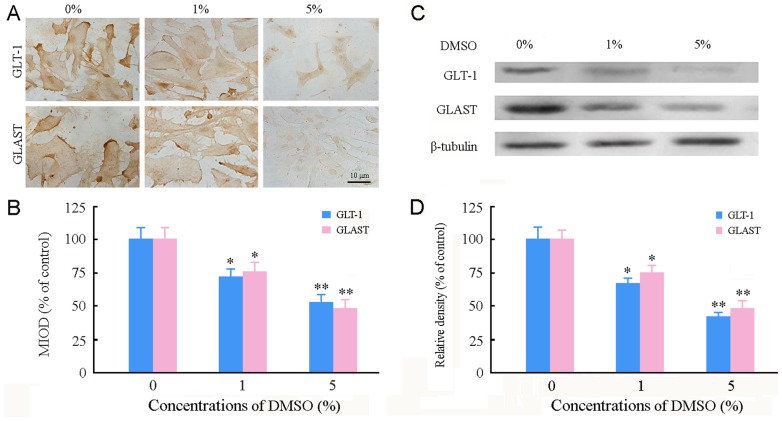
Effects of DMSO on expression of GLT-1 and GLAST in cultured mouse cortical astrocytes. (**A**) Representative micrographs showing immunoreactivity of GLT-1 and GLAST in astrocytes treated with various concentrations of DMSO for 24 h. (**B**) Mean integrated optical density (MIOD) of immunostaining for GLT-1 and GLAST. (**C**) Representative western blot bands showing expression levels of GLT-1 and GLAST in astrocytes. (**D**) Quantitative analysis revealed that GLT-1 and GLAST protein levels were decreased in astrocytes treated with DMSO in a dose-dependent manner. Data are shown as a mean ± SEM of five (for immunostaining) or four (for Western blot) independent experiments performed in triplicate. *P<0.05 and **P<0.01 versus control group.

Astrocytes are responsible for maintaining glutamate homeostasis in the central nervous system by glutamate transporters that are vulnerable to oxidative stress [37]. Thus, we examined whether increased ROS generation resulted in down-regulation of GLT-1 and GLAST in DMSO-treated astrocytes. The immunoreactivity for these proteins decreased in astrocytes exposed to 1% and 5% DMSO (Figure 7A–B). DMSO-reduced GLT-1 and GLAST expression in a concentration-dependent pattern was confirmed by the Western blot analysis (Figure 7C–D).

## Discussion

DMSO at concentrations of 0.5–1.5% is widely used as a solvent for various pharmacological agents in both cell culture and *in vivo* studies [3]. However, the biological effects of DMSO may be neglected, because a vehicle control is not always incorporated into the design of experiments. An early study suggested that exposure of primary hippocampal cell cultures to DMSO in the range of 0.5–1.0% for 24 h has no significant effect on the survival of neurons, but can prevent glutamate-induced neuronal death via suppression of NMDA- and AMPA-induced ion currents and calcium influx [38]. A followed study has shown that treatment with DMSO at concentrations of 0.5% or 1.0% for 6 DIV produces a substantial decrease in the number of cultured hippocampal neurons [12]. In the present study, a DMSO concentration of 1% does not significantly affect the astrocyte survival during a 24-h exposure period, but impairs cell viability, mitochondrial integrity and glutamate transporter expression. Exposure of astrocyte cultures to DMSO at concentrations of 5% significantly impairs cell survival and increased apoptosis. Collectively, these results provide strong *in vitro* evidence for the neuropharmacological and neurotoxicological effects of DMSO.

The amphiphilic characteristic of DMSO imparts extensive actions in membrane fusion processes, cryopreservation, and membrane permeability enhancement [39], [40]. A molecular dynamics simulation employing coarse-grained models suggested that DMSO modulates the structure and properties of cell membranes in a concentration-dependent manner [27]. DMSO induces membrane thinning and increases fluidity of the membrane's hydrophobic core at low concentrations (<10 mol%), formation of transient water pores in the bilayer membrane at 10–20 mol%, and local loss in integrity of the phospholipid membranes at higher concentrations. The present results demonstrated that cultured mouse astrocytes exposed to 1.0% DMSO for 24 h exhibit impairments of the mitochondrial integrity and ΔΨm, although their general growth profile is not altered compared to those in control medium. This result suggests that mitochondrial membrane is vulnerable to DMSO, which might be due to its relatively high membrane fluidity [28].

Mitochondria are the center of cell metabolism and energy transformation; and their malfunction decreases cell viability [29], [41]. In agreement with this notion, we demonstrated that DMSO inhibits astrocyte viability in a dose-dependent manner, accompanied with mitochondrial structural and functional disruption. Astrocytes exposed to DMSO at concentrations of 1.0% also increases Cyt *c* release and activated caspase 3 expression, and decreases anti-apoptotic protein Bcl-2 expression, supporting the review that rupture of mitochondria is an initial trigger of apoptotic cascades [28], [35]. Furthermore, the present results indicated that DMSO dose-dependently increases mitochondria-derived ROS production, which is consistent with the notion that degenerated mitochondria are the primary site of ROS production [32]. Taken together, these results reveal that mitochondrial impairment is a primary event in the astrocyte toxicity of DMSO.

Glutamate, the major excitatory amino acid neurotransmitter in the brain, can become potentially toxic when it over-accumulates in the synaptic space [42]. As mentioned earlier, astrocytes are responsible for maintaining brain glutamate homeostasis via glutamate transporters [24]. Oxidative stress inhibits glutamate transporter expression and function, which has been implicated as a main pathogenesis for glutamate excitotoxicity in a variety of pathological conditions, including brain ischemia [43], [44], traumatic brain injury [45], epilepsy [46] and neurodegeneration [47], [48]. The present results have demonstrated that DMSO causes down-regulation of GLT1 and GLAST in cultured astrocytes. In addition to increased ROS production, decreased cell viability and mitochondrial dysfunction may impair glutamate transporter synthesis by astrocytes. High concentration of DMSO has been shown to degrade membrane structure and disturb secondary protein structures within membrane proteins [26], [27]. This may also contribute to decreased expression of GTL1 and GLAST. Down-regulation of glutamate transporters may lead to neuronal excitotoxicity, thus exacerbating DMSO-induced neuronal damage. The results help to explain the occurrence of epilepsy in humans [14]–[18] and rats [49] following exposure to high concentration of DMSO.

In summary, the present results reveal that mitochondrial damage, oxidative stress and apoptosis are involved in DMSO-induced astrocyte toxicity. DMSO at concentrations of 1% impairs cell viability, mitochondrial integrity and glutamate transporter expression of astrocytes, although does not affect their survival and apoptosis during a 24-h exposure period. Further *in vivo* studies are necessary to address whether astrocyte dysfunction is involved in neurobehavioral consequences of acute high dose or chronic low dose exposure to DMSO. Exploring these issues will help objective evaluation of pharmacological or toxicological effects of DMSO itself, or as a solvent or carrier for hydrophobic agents.

## Experimental Methods

### Animals

Adult CD1 mice (8–10-weeks-old) weighting 20–25 g were housed 4 per cage in a room maintained at 22±1°C with an alternating 12 h light–dark cycle. Food and water was available ad libitum. Animal protocols used in the study were approved by the Institutional Animal Care and Use Committee (IACUC) of Nanjing Medical University.

### Astrocyte cultures and drug treatment

The cerebral cortices of post-natal day 1–3 CD1 mice were isolated and dissociated with 0.125% trypsin (Millipore, Billerica, MA, USA) digestion and trituration with a flame-polished pasture pipet. Following filtration, dissociated cells were suspended in Dulbecco's modified Eagle's medium (DMEM) plus 10% fetal bovine serum (FBS) (Gibco BRL, Guithersburg, MD, USA), then plated into tissue culture flasks coated with 100 mg/ml poly-L-lysine (Sigma-Aldrich, St. Louis, MO, USA), and placed in a humidified cell culture incubator at 37°C in an atmosphere of 95% air and 5% CO_2_. Culture medium was changed every 2 or 3 days. Seven or eight days after plating, cells were passaged, subjected to a partial media exchange, and incubated for another 5–6 days. The cultures consisted of at least 95% astrocytes as determined by immunostaining against the astrocyte marker glial fibrillary acidic protein (GFAP) and the microglial marker CD11b.

Astrocytes of the second passage were then resuspended in DMEM/10% FBS, and plated into poly-L-lysine-coated plates as follows: 24-well microtiter plates (5×10^5^ cells/well) for morphological, immunocytochemical, ΔΨm and apoptosis assays, 96-well microtiter plates (2×10^4^ cells/well) for viability assay, and 6-well plates (2×10^6^ cells/well) for Western blot and flow cytometric analyses. The cells were allowed to grow for 1–2 days to reach confluence, then treated with different concentrations (0, 1 and 5%) of DMSO (Sigma-Aldrich, St. Louis, MO, USA) for 24 h.

### Astrocyte survival and morphological assay

After treatment with different concentrations of DMSO for 24 h, the growth of astrocytes was examined by a phase contrast microscope and 5 randomly selected areas per well were imaged at 100×magnification. Total number of cells in each image was counted using the Image J software (National Institutes of Health, Bethesda, MD, USA). The density of cells (cell number per mm^2^) was calculated and expressed as a percentage of the untreated control cells.

### Cell viability assay

Cell viability was evaluated by MTT assay [50]. After replacement of fresh DMEM, 20 µl of 5 mg/ml MTT (Sigma-Aldrich) was added and incubated for 4 h, the culture medium was discarded and 150 µl DMSO was used to dissolve the precipitate. The absorbance was measured at 490 nm using an Automated Microplated Reader ELx800 (BioTek, Winooski, VT, USA). Results are expressed as percentage of control values.

### Transmission electron microscopy

DMSO-treated astrocytes were fixed with 4°C 2.5% glutaraldehyde in phosphate buffer saline (PBS, pH 7.4) for 2 h. The cells were detached with 0.125% trypsin, and collected by centrifugation for 10 min at 1000 *g*, rinsed with PBS and post-fixed in 1% osmium tetroxide with 0.1% potassium ferricyanide for 48 h. The cell samples were dehydrated through a graded series of ethanol, and embedded in Epon 812. Sixty-micrometer-thick ultrasections were stained with 1% uranyl acetate and 0.1% lead citrate, and examined with a Jeol 1200EX electron microscope (Jeol Ltd., Akishima, Tokyo, Japan). Ten astrocytes from each group were randomly captured at a magnification of ×12000 and used for quantitation of mitochondrial cross-sectional area using an Image-Pro Plus 6.0 Analysis System (Media Cybernetics Inc., San Francisco, CA, USA). The percentage of vacuolated mitochondria, characterized by partial or complete loss of cristae, was counted.

### Measurement of ΔΨm

ΔΨm was assessed using a live cell assay with the fluorescent lipophilic cationic dye TMRE (Invitrogen, Karlsruhe, Germany). This dye is a cell permeant, positively-charged, red-orange dye that readily accumulates in active mitochondria due to their relative negative charge [51]. After treatment with different concentrations of DMSO for 24 h, cultured astrocytes were stained with 200 nM TMRE for 30 min at 37°C, then washed twice in medium and re-suspended in PBS. The fluorescent images were immediately taken using a Zeiss AX10 inverted fluorescence scope (Carl Zeiss Microimaging Inc., Germany). Some samples incubated with 20 µM carbonylcyanide-p-trifluoromethoxyphenylhydrazone (FCCP), an uncoupler of electron transport and oxidative phosphorylation, for 10 minutes prior to staining with TMRE, served as a positive control for depolarized mitochondria. The TMRE fluorescence density was analyzed using an Image-Pro Plus 6.0 Analysis System. The data are presented as percentage of control values.

### Assay of Cyt *c* release

After treatment with different concentrations of DMSO for 24 h, cultured astrocytes were incubated in 4°C permeabilisation buffer (100 mM KCl, 100 µg/ml digitonin in PBS) for 3–5 min. The cells were fixed with 4% paraformaldehyde in PBS for 20 min at room temperature, washed twice with PBS, and detached enzymatically. The cells were then centrifuged, resuspended in 1 ml blocking buffer (3% bovine serum albumin, 0.05% saponin in PBS) for 1 h at room temperature, and incubated with mouse monoclonal anti-*Cyt c* (1∶2000; Abcam, San Francisco, CA, USA) overnight at 4°C. The cells were rinsed twice with PBS, then incubated with FITC conjugate secondary antibody (1∶200, Vector Laboratories, Burlingame, CA, USA) for 1 h at the room temperature. The samples were analyzed by a FACSCalibur flow cytometer (Becton-Dickinson) at an excitation wavelength of 488 nm and an emission wavelength of 525 nm.

### Measurement of mitochondrial and intracellular ROS generation

Mitochondria-mediated ROS generation was detected with the mitochondrial superoxide indicator MitoSOX-Red. Intracellular ROS were detected using an oxidation-sensitive fluorescent probe 2′-7′-dichlorodihydrofluorescein diacetate (DCFH-DA). DCFH-DA was deacetylated intracellularly by nonspecific esterase, which was then further oxidized by ROS to the fluorescent compound 2,7-dichlorofluorescein (DCF) [52]. Astrocytes were treated with different concentrations of DMSO (0, 1 and 5%). Cells were then washed twice in PBS and placed in a humidified incubator (5% CO_2_ and 95% air) at 37°C with 5 mmol/L of MitoSOX-Red (Invitrogen) for 15 min or 2.5 µg/ml DCFH-DA (Sigma-Aldrich) in PBS for 30 min. Subsequently, the cells were washed twice in culture medium and trypsinized. The cell pellet was re-suspended in PBS followed by analysis on a FACSCalibur flow cytometer (Becton-Dickinson).

### Isolation of mitochondrial fraction and cytosolic fraction

Each cell sample was homogenized in ice-cold lysis buffer containing 200 mM mannitol, 80 mM HEPES-KOH (pH 7.4), and the protease inhibitor cocktail. Homogenates were centrifuged briefly at 750 rpm for 10 min at 4°C in an Eppendorf centrifuge. After removing the top half of the supernatants, the rest of the supernatants were centrifuged at 8,000 rpm for 20 min at 4°C. The pellets were then washed 3 times and suspended in the lysis buffer as the mitochondrial fraction. Supernatants of 8,000 rpm centrifugation were centrifuged at 100,000 rpm for 1 h at 4°C. The resulting supernatants were cytosolic fraction. The protein concentration of each sample was determined using the Bradford assay.

### Western blot analysis

DMSO-treated astrocytes were washed with Ca^2+^- and Mg^2+^-free PBS, then detached from the plates by scraping, and harvested by centrifugation for 5 min at 1500 *g*. Cell pellets were suspended in 60 µl lysis buffer containing 200 mM phenylmethylsulfonylfluoride (PMSF) and homogenized on ice by an ultrasonic homogenizer. The lysates were centrifuged (12,000 rpm for 15 min at 4°C) and the protein concentration in the extracts was determined using the Bradford assay. Samples of the cell extracts or mitochondrial and cytosolic fractions were denatured with a SDS sample buffer and separated by 10% SDS-PAGE. Proteins were transferred onto PVDF membranes followed by blocking membranes with 5% skimmed milk dissolved in TBST (50 mmol/L Tris-HCl, pH 7.5, 150 mmol/L NaCl, 0.1% Tween20) at room temperature for 2 h. After three washes with TBST buffer, membranes were incubated with mouse monoclonal anti-*Cyt c* (1∶2000; Abcam), rabbit polyclonal antibody against procaspase 3 (1∶1200; Abcam), rabbit polyclonal antibody against cleaved-caspase 3 (1∶800; Abcam), rabbit polyclonal antibody against Bcl-2 (1∶200; Santa Cruz BioTech, Santa Cruz, CA, USA), rabbit polyclonal antibody against GLT-1 (1∶500; Santa Cruz BioTech) or rabbit polyclonal antibody against GLAST (1∶500, Santa Cruz BioTech), rabbit polyclonal antibody against COX IV (1∶1200; Abcam) or mouse monoclonal antibody against β-tubulin (1∶3000; Abcam) at 4°C overnight. The bands were detected with biotin conjugated secondary antibodies (1∶200, Vector Laboratories), rinsed and visualized by ECL plus Western blotting detection system. β-tubulin was used as an internal control.

### Apoptosis assay

Cell apoptosis was detected by TUNEL assay using the In Situ Cell Death Detection Kit (Roche Diagnostics GmbH, Mannheim, Germany). After treatment with different concentrations of DMSO, astrocytes on coverslips were washed with PBS and fixed in freshly made 4% paraformaldehyde for 1 h. After permeabilization with 0.1% Triton X-100 (dissolved in 0.1% sodium citrate) for 5 min, the coverslips were incubated with 20 µg/ml proteinase K for 15 min at 37°C, then incubated with 100 µl TUNEL reaction mixture for 1 h at 37°C. The reaction was ceased by washing with PBS for 3 times. After counterstaining with Hoechst 33342 (Sigma-Aldrich), TUNEL positive cells were captured using a Leica DM4000B digital microscope (Leica Microsystems, Wetzlar, Germany). The percentage of TUNEL positive astrocytes in the culture was counted.

### Immunostaining

After washing 3 times in PBS, astrocytes on coverslips, or dewaxed hippocampal sections, were treated with 2% Triton X-100 containing 0.5% bovine serum albumin (BSA) (Sigma-Aldrich) for 1 h. They were then incubated with mouse monoclonal antibody against GFAP (1∶1000; Sigma-Aldrich), rabbit polyclonal antibody against GLT-1 (1∶400; Santa Cruz) or rabbit polyclonal antibody against GLAST (1∶400, Santa Cruz) at 4°C overnight. After PBS rinses, appropriate secondary antibodies were applied for 1 h at 37°C. The signals were visualized by 3,3′-diaminobenzidine (DAB) (Sigma-Aldrich) and captured using a Leica DM4000B digital microscope. The mean integrated optical density (MIOD) was measured to assess the expression levels of GFAP, GLT-1 or GLAST using Image J software. The data are presented as percentage of control values.

### Statistical analysis

Data are expressed as mean ± SEM. All statistical analyses were performed using SPSS software, version 16.0 (SPSS Inc., USA). The significance of difference was evaluated with one-way analysis of variance (ANOVA) procedures followed by the Bonferroni test. *P*-values less than 0.05 were considered statistically significant.
